# Overview of Multi-Scale Simulation Techniques for Three Typical Steel Manufacturing Processes

**DOI:** 10.3390/ma17133173

**Published:** 2024-06-28

**Authors:** Cheng-Hui Xia, Kaiyang Wang, Xuexia Song, Weiming Pan, Wei Li, Hong-Hui Wu, Kun Dou, Yuantao Xu, Zelin Tong, Shaojie Lv, Jingzhou Lu, Shuize Wang, Wanlin Wang, Xuejun Jin, Xinping Mao

**Affiliations:** 1 Shanghai Key Laboratory of Materials Laser Processing and Modification, Shanghai Jiao Tong University, Shanghai 200240, China; 2 Beijing Advanced Innovation Center for Materials Genome Engineering, University of Science and Technology Beijing, Beijing 100083, China; 3 School of Metallurgy and Environment, Central South University, Changsha 410083, China; 4 National Center for International Research of Clean Metallurgy, Central South University, Changsha 410083, China

**Keywords:** cross-scale simulation, casting, hot rolling, laminar cooling

## Abstract

Steel products typically undergo intricate manufacturing processes, commencing from the liquid phase, with casting, hot rolling, and laminar cooling being among the most crucial processes. In the background of carbon neutrality, thin-slab casting and direct rolling (TSCR) technology has attracted significant attention, which integrates the above three processes into a simpler and more energy-efficient sequence compared to conventional methods. Multi-scale computational modeling and simulation play a crucial role in steel design and optimization, enabling the prediction of properties and microstructure in final steel products. This approach significantly reduces the time and cost of production compared to traditional trial-and-error methodologies. This study provides a review of cross-scale simulations focusing on the casting, hot-rolling, and laminar cooling processes, aiming at presenting the key techniques for realizing cross-scale simulation of the TSCR process.

## 1. Introduction

**Figure 1:**
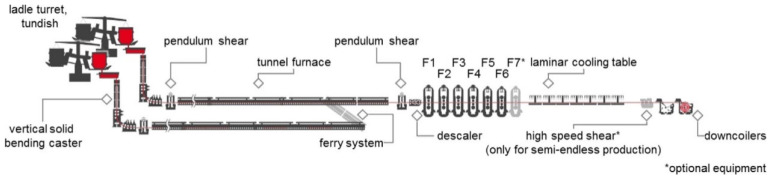
A typical arrangement of a compact CSP plant with two strands. Reprinted with permission from [2].

Steel products usually undergo complex manufacturing processes starting from the liquid metal, in which casting, hot-rolling, and cooling phase transition processes are the most important. Under the background of carbon neutrality, the high requirement of low-carbon emissions brings tremendous pressure to the steel industry. As one of the representative near-net-shape steel manufacturing technologies, thin-slab casting and direct rolling (TSCR) technology has received considerable attention, which involves the above three processes but is simpler and more energy-efficient compared with the conventional processes [1,2]. A typical arrangement of a two-strand Compact Strip Production (CSP) plant is represented in Figure 1 [2].

Within the realm of steel design and enhancement, there is a growing utilization of computational modeling and simulation to forecast the microstructure and properties of steel items. The computational method can reduce the time and cost of production compared to traditional trial-and-error methodologies. Implementing cross-scale simulations of the TSCR process helps improve the efficiency of TSCR technology.

The TSCR process commences with continuous casting, where high-temperature molten steel transitions from liquid to solid. This procedure involves three stages: initial cooling within the mold, followed by a secondary cooling zone, and concluding with an air-cooling zone. The simulation of the solidification process of continuous casting involves multi-physical fields with cross-scale problems, which can be mainly divided into macroscopic, mesoscopic, and microscopic scales [3].

After casting, conventional slabs undergo cooling, followed by reheating, before hot rolling. Conversely, with TSCR technology, the hot thin slab is immediately rolled following homogenization in a tunnel furnace, achieving significant energy savings. The rolling process entails a thermomechanical–metallurgical phenomenon, presenting a challenge for interdisciplinary coupling in rolling simulation [2,4].

After the hot-rolling process, the slab progresses to the laminar cooling and curling stage. Besides the external deformation on the outside of the slab, this process also induces internal solid-state transformations, such as the conversion of austenite (γ) into ferrite (α)/pearlite. The numerical simulation of the laminar cooling involves complex challenges, requiring the integration of temperature, composition, and strain fields, among other multi-physical field interactions. Furthermore, the complex interplay between micro-, meso-, and macro-scales in this process presents significant challenges for cross-scale simulation studies, underscoring the necessity for advanced computational methods to accurately model and forecast outcomes [5,6,7]. Considering the curling process only involves the subsequent deformation process without phase change generally, it will not be addressed in the present study.

In this study, we examine the use of cross-scale simulation in the casting, hot-rolling, and laminar cooling processes, with a focus on providing key techniques for achieving successful simulations of the TSCR process. Section 2, Section 3, and Section 4 discuss the essential cross-scale simulation techniques for casting, hot-rolling, and laminar cooling processes, respectively. In Section 5, we summarize these techniques and provide an outlook for future developments.

## 2. Cross-Scale Simulation of the Continuous Casting Process

Continuous casting of steel stands as a cornerstone in the steel production industry, providing a robust method for efficiently and precisely manufacturing high-quality steel products. The process involves the solidification of molten steel into semi-finished products, such as slabs, blooms, or billets, which are subsequently processed to manufacture various steel products. Achieving control over the complex phenomena occurring during continuous casting requires an in-depth understanding of the process at multiple scales. At the macroscopic level of continuous casting, heat, momentum and mass transfer, and multi-physics field coupling are mainly considered [8,9,10,11,12]. To describe the macroscopic solidification process, it is mainly modeled and coupled with various finite element (FE) software, such as ProCAST, ANSYS, and FLUENT, for predicting the heat transfer, flow, mass transfer, shrinkage, and cracking of the steel solidification process [13,14,15,16,17,18,19]. However, the macroscopic simulation can only obtain the changes in temperature and flow rate during the solidification process and cannot predict the solidification characteristics of castings, such as grain size and nucleation number. On the mesoscopic and microscopic scales, the solidification process manifests itself in the nucleation and growth of grains [20,21,22], which determine the microscopic solidification structure and, subsequently, influence the material properties. Solidification structure simulation can acquire important details, such as solid–liquid interface motion, crystal morphology, size distribution, and microscopic defects. To describe the microscopic solidification process, there are the Monte Carlo (MC) method, the Cellular Automata (CA) method, and the Phase-Field (PF) method, etc. [23].

### 2.1. Microscopic/Mesoscopic Solidification Simulations

In this section, we provide a brief introduction of the microscopic simulation methods, namely MC, CA, and PF methods. Please refer to Section 4 for the formulas of these methods, as the solidification and cooling processes of steels primarily involve phase transition and diffusion phenomena.

#### 2.1.1. Application of the Monte Carlo Method in Solidification Simulations

The MC method serves as a powerful numerical technique, extensively used in solidification simulations to model the complex phenomena occurring during the transition from liquid to solid state in materials, such as metals and alloys [24]. This method relies on random sampling and statistical analysis to model the microstructure evolution during solidification processes.

In the context of solidification, the MC method is employed to predict the nucleation and growth of crystals [25], the formation of grain structures [26], and the evolution of phases within a material as it cools and solidifies [27]. By simulating the random movement of atoms or particles within a system, MC simulations can capture the stochastic nature of solidification phenomena, such as nucleation site selection, grain boundary movement, and impurity segregation. One of the key strengths of the MC method in solidification simulations is its ability to model the inherent randomness and complexity of solidification processes, enabling researchers to study the effect of different factors, such as cooling rates, alloy composition, and thermal gradients on microstructure development.

By running multiple iterations of the simulation with randomized inputs, MC simulations provide insights into the probabilistic distribution of microstructural features and can forecast the ultimate properties of the solidified material. Furthermore, the MC method allows researchers to explore different solidification scenarios, optimize process parameters, and design novel materials with tailored microstructures and properties. Its flexibility and scalability make it a valuable tool for investigating phase transformations, grain growth behavior, and defect formation mechanisms in solidifying materials [28].

In summary, the MC method is a versatile and powerful tool in solidification simulations, offering a probabilistic approach to understanding and predicting the complex microstructural evolution that occurs during the solidification of materials, such as metals and alloys. However, this method overlooks the physical mechanisms of grain growth, such as grain orientation preferences, and relies solely on energy changes without explicitly incorporating the crucial factor of solidification time. As a result, the MC method can only provide qualitative, rather than quantitative, analysis of the influence of different physical phenomena on the crystallization process.

#### 2.1.2. Application of the Cellular Automata Method in Solidification Simulations

The CA method offers a robust tool for simulating solidification processes in materials, such as steel [29]. Within the context of metallurgy, CA models provide a detailed and dynamic way to study the evolution of microstructures during solidification. In the CA method, the material is partitioned into cells that interact with neighboring cells based on predefined rules. Each cell undergoes state changes over discrete time steps, mimicking the progression of solidification. By considering factors such as nucleation, growth kinetics, and thermal gradients, CA models capture the complex phenomena occurring during solidification.

One of the key advantages of using CA for solidification studies is its ability to simulate grain growth, dendrite formation, and other microstructural features with high spatial resolution [30]. This fine-grained approach allows researchers to scrutinize the effects of different parameters on microstructure evolution, such as cooling rates, alloy composition, and impurity content.

Moreover, CA models can be coupled with other computational methods [31], such as PF or FE methods, to create more comprehensive simulations of solidification processes. This integration enables researchers to explore the interactions between macroscopic thermal conditions and microscopic crystal growth in greater detail.

**Figure 2:**
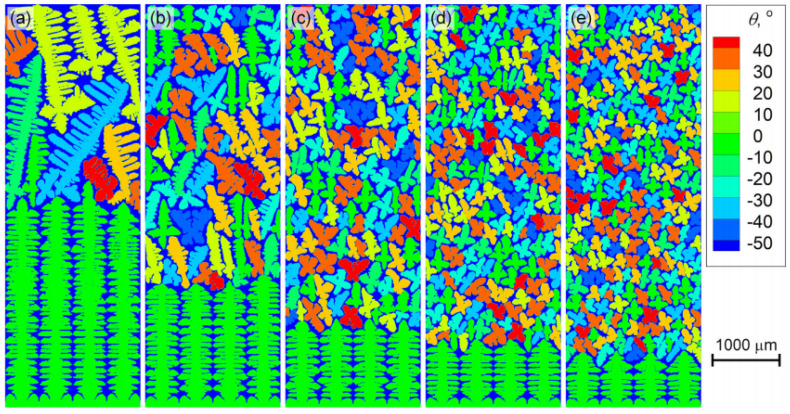
Effect of the cooling rate on the solidification morphology of the Fe-0.82 C alloy at the thermal gradient of 3000 K/m: (**a**) 0.25 K/s, (**b**) 0.40 K/s, (**c**) 0.60 K/s, (**d**) 0.80 K/s, and (**e**) 1.00 K/s. Reprinted with permission from [32].

Wang et al. [32] formulated a solidification model incorporating dendritic growth and heterogeneous nucleation to explore the columnar to equiaxed transition (CET) behavior of Fe-C alloys at the microscopic level and assess the effect of the cooling rate on the CET location. Figure 2 shows the dendritic structures at the solid fraction of 0.7 of the Fe-0.82C alloy under a thermal gradient of 3000 K/m, with the cooling rate ranging from 0.25 to 1.00 K/s. As the cooling rate rises, the CET is accelerated, largely retaining the columnar-tip type morphology. Additionally, there is a rise in the number of newborn dendrites, a reduction in their size, and a transformation of the inner dendrites from the columnar-equiaxed mixed type to the equiaxed type.

#### 2.1.3. Application of the Phase-Field Method in Solidification Simulations

PF methods rooted in thermodynamics are often used to simulate the phase transition and the evolution of the material microstructures at the microscopic level. By introducing the diffusion interface method, the PF simulation can effectively characterize the phase interface thickness and provide continuous-order parameter evolution in the model, which solves the problem that it is difficult to track the interface motion of the traditional method [33,34].

In the realm of solidification processes, the PF model stands out as a potent tool for understanding and simulating complex solid–liquid interface dynamics. This approach has revolutionized the study of solidification phenomena by providing a detailed and computationally efficient framework to analyze phase transformations, microstructure evolution, and pattern formation during material solidification. Through solving coupled partial differential equations derived from energy minimization principles [35], PF simulations track the evolution of these interfaces over time, revealing insights into the nucleation, growth, and coarsening processes involved in solidification [36,37,38,39].

The versatility of the PF model allows researchers to explore a wide range of solidification phenomena, from dendritic growth and grain refinement to complex morphological transitions in alloy solidification. By adjusting model parameters and incorporating thermodynamic and kinetic data, the PF approach facilitates the investigation of various materials, processing conditions, and geometric configurations, offering predictive capabilities for optimizing solidification processes in industries, such as metallurgy, casting, and additive manufacturing [36].

**Figure 3:**
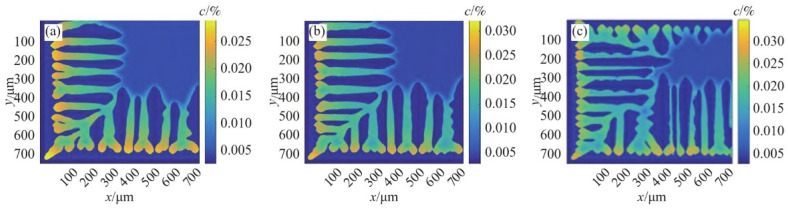
The change in the columnar crystal structure formed through directional solidification under various anisotropy coefficients: (**a**) 0.04, (**b**) 0.05, and (**c**) 0.065. Reprinted with permission from [40].

**Figure 4:**
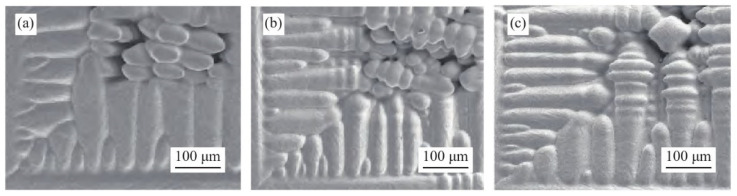
Observation of the directional solidification-induced growth morphology of Fe-C alloy columnar dendrites via scanning electron microscopy: (**a**) weak anisotropy coefficient, and strong anisotropy coefficient under low (**b**) and high (**c**) undercooling conditions. Reprinted with permission from [40].

Zeng et al. [40] integrated the PF model with flow fields to simulate the dendrite growth in the directional solidification of Fe-C alloys. They investigated how anisotropy coefficients affect dendrite growth and dendrite behavior under forced convection. The simulation results of the solute field of the solidified column at the same calculation time step are shown in Figure 3. The dendrite size grows with the increasing anisotropy coefficient and becomes denser. It is also found that the root isotropic crystals are significantly refined as the anisotropy factor increases, and the smooth dendrite wall surface is flooded with more bumps, which tends to develop into secondary dendrites. The dendrite growth morphology observed experimentally through optical microscopy in Figure 4 generally aligns with the simulation findings.

### 2.2. Cross-Scale Simulations

As described above, single-scale microscopic simulation methods can achieve precise simulations of solidification organization. However, the actual cross-section dimensions of the continuous casting billet, spanning tens of centimeters to several meters in length and width, exceed the capabilities of microscopic simulation. Microscopic simulations using the CA or PF method demand significant computational resources and time, often beyond current capabilities. Thus, coupling macroscopic heat transfer, mass transfer, flow, and microstructure simulations becomes essential.

The FE method, or finite element analysis (FEA), employs mathematical approximations to simulate real physical systems, simplifying complex problems into manageable ones. It divides the solution domain into interconnected subdomains, known as finite elements, assigns an approximate solution to each unit, and then deduces the overall satisfaction condition of the domain to solve the problem effectively. The FE method boasts not only high computational accuracy but also the ability to adapt to various complex shapes, rendering it an effective tool for macroscopic engineering analysis.

The cross-scale CA and finite difference (FD)/FE coupling model (CA-FD/FE) is widely used in solidification research. Macroscopic and microscopic calculations employ distinct grid sets, with the macroscopic simulations of heat transfer utilizing coarser meshes. This approach enables faster computation and extraction of the primary characteristics of diverse physical fields. The microstructure simulation subdivides based on the macroscopic simulation grid, employing a finer grid for detailed analysis and inheriting the results of the macroscopic simulation for further computation. This approach leads to more accurate results within a shorter timeframe. With the CA-FD/FE model offering substantial benefits in multi-scale and multi-field coupling studies, and its integration into the commercial software ProCAST, it provides a convenient and efficient tool for scientific research and exploration. This model has been widely used and has evolved progressively, moving from conventional casting to continuous casting processes [41,42].

The following pair-wise mixture model is used to calculate the thermophysical properties at different temperatures, including density, thermal conductivity, and enthalpy, etc. [41]:

(1) $$P=\sum_{i}X_{i}P_{i}+\sum_{i}\sum_{j>i}X_{i}X_{j}\sum_{s}\Omega_{s}{\left(X_{i}-X_{j}\right)}^{s},$$

where *P* and *P_i_* are the thermophysical properties of one phase and pure element, respectively, Ω*_s_* is the binary interaction parameter, and *X_i_* is the mole fraction of the element *i*. The calculation results of *P* will be applied in the CA-FE model of modeling the solidification structure, which mainly involves the heat transfer model, nucleation model, and dendrite-tip growth kinetics.

An unsteady-state heat transfer equation is available as:

(2) $$k\nabla^{2}T-\tilde{\rho }c_{p}\dot{T}=0,$$

where k is the heat conductivity, $\tilde{\rho }$ is the density, and $c_{p}$ is the specific heat. The calculation incorporates the evolution of latent heat during solidification through the effective specific heat method:

(3) $$c_{p}^{\prime }=c_{p}-L\left(\frac{df_{s}}{dT}\right),$$

where $c_{p}^{\prime }$ is the effective specific heat, *L* the latent heat and $f_{s}$ the solid fraction. A continuous nucleation distribution function,

(4) $$\frac{dn}{d\left(\Delta T\right)}=\frac{n_{\max}}{\sqrt{2\pi }\Delta T_{\sigma }}\exp\left[-\frac{1}{2}{\left(\frac{\Delta T-\Delta T_{\max}}{\Delta T_{\sigma }}\right)}^{2}\right],$$

is applied to depict the alteration in grain density. Δ*T* is the calculated local undercooling, K; Δ*T*_max_ is the mean undercooling, K; Δ*T*_σ_ is the standard deviation, K, and *n*_max_ represents the highest achievable nucleation density when all the nucleation sites are activated during cooling, m^−3^. A mathematical-fitted KGT (Kurz, Givoanola, and Trivedi) model is used to describe the dendrite growth:

(5) $$V\left(\Delta T\right)=a_{2}\Delta T^{2}+a_{3}\Delta T^{3},$$

where *a*_2_ and *a*_3_ are the fitting parameters, which reflect the growing velocity of the dendrite tip.

**Figure 5:**
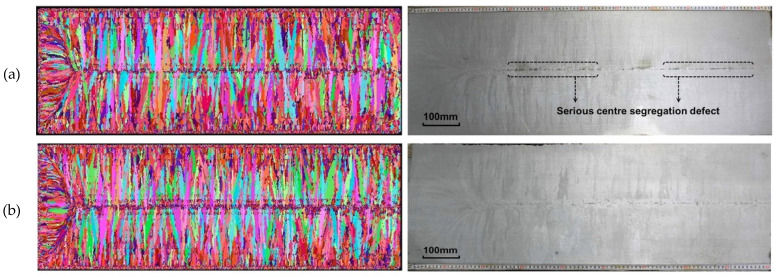
Comparison of simulation (**left**) and experimental (**right**) results before (**a**) and after (**b**) optimizing the micro-alloying elements’ contents and the continuous casting process parameters. Reprinted with permission from [43].

**Figure 6:**
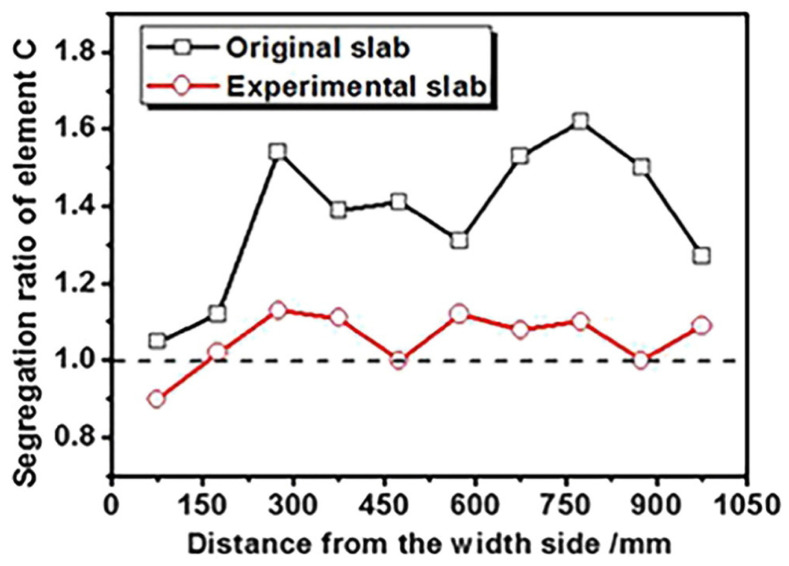
Comparison of the segregation ratios of element C between the initial slab and experimental slab [43].

Gao and Yang [43] established CA-FE and secondary dendritic arm spacing (SDAS) models to investigate the macrostructure of slab cross-sections and investigated the effects of superheat degree and casting speed on the macrostructure. In Figure 5, the results from both the simulation and experiment distinctly delineate three structural zones, i.e., the outer chill zone, the columnar zone, and the interior equiaxed zone. Following adjustments to the micro-alloy element content and experimental process parameters, the central equiaxed crystal rate of the slab cross-section increased by 8.5%, and central segregation defects in the experimental slab were enhanced. As depicted in Figure 6 the original billet had severe positive segregation, with an average segregation ratio of 1.37. The adjusted slab also had slight positive segregation, with an average segregation ratio of 1.05. Hence, the adjusted billet can effectively decrease the likelihood of center segregation defects.

**Figure 7:**
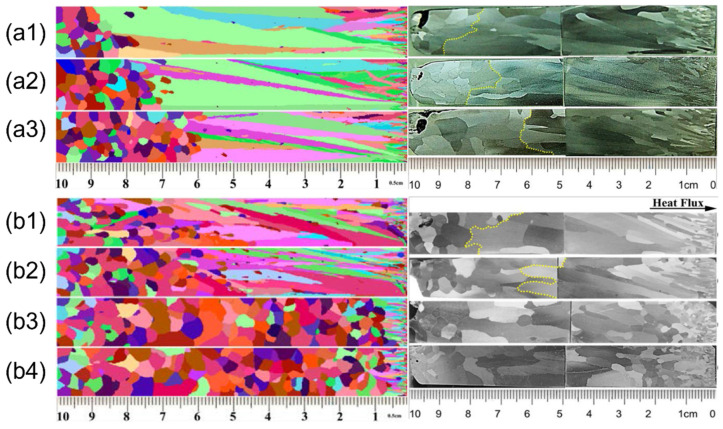
Effect of different superheats and cooling rates on the macrostructure of cast billets (the left is the simulation results, and the right is the experimental results): (**a1**) high cooling rate, (**a2**) middle cooling rate, (**a3**) low cooling rate, (**b1**) 40 °C, (**b2**) 30 °C, (**b3**) 20 °C, and (**b4**) 10 °C. Reprinted with permission from [44].

Bai et al. [44] developed thermal simulation equipment (TSE) to imitate the solidification process in the industrial continuous casting of slabs, while the temperature distribution, microstructure evolution, and CET transition were simulated by the CA-FE model. The experimental findings indicated that the device can effectively regulate the temperature gradient and grain growth rate of the samples. The upper part of Figure 7 shows the increase in the center equiaxed crystal ratio with the decreasing superheat temperature. At superheat levels of 40 °C and 30 °C, the central equiaxed crystal ratio of the samples was around 26% and 42%, respectively. Conversely, at superheat levels of 10 °C and 20 °C, the samples showed no discernible formation of columnar grains. In the lower part of Figure 7, it is evident that reduced cooling rates led to increased proportions of center equivalent crystals in the samples. Specifically, as the cooling rate decreased, the center equiaxed crystal ratios were 14%, 23%, and 42%, respectively.

Li et al. [45] utilized the CA-FE model to imitate the solidification organization of thin slabs during high-speed continuous casting. Their simulations closely matched the solidification organization observed in actual steel samples. Additionally, they thoroughly investigated the impacts of continuous casting process parameters and alloying elements. The optimal process parameters were determined as follows: a drawing speed of 5.2~5.4 m·min^−1^, a superheat of 20~25 °C, and a specific water flow of 1.62~1.64 L kg^−1^. Simultaneously, the Si content in the thin slab was strategically raised to refine the grain size and enhance the production efficiency. This research offers valuable theoretical insights for thin-slab production, enhancing the efficiency of continuous casting, and minimizing occurrences.

As mentioned above, the CA-FD/FE model realizes the coupling calculation from the macroscopic-scale multi-physical field to the microscopic-scale grain structure, which can simulate the microscopic segregation, columnar crystal, equiaxed crystal, tissue distribution, and CET of alloy solidification under different preparation methods and process conditions, and provide guidance and basis for solidification process and tissue structure control.

## 3. Cross-Scale Simulation of the Hot-Rolling Process

The difference between the traditional and TSCR processes mainly comes from the hot-rolling process, because the microstructure of the slab with coarse austenite grains does not undergo γ-α-γ transformation before hot rolling within the TSCR process. Due to the products finally undergoing phase transition from high-temperature austenitic inherited from the hot-rolling process, the room-temperature state of products produced by the TSCR process is highly relevant to the process of hot rolling. For example, rolling the slab below the recrystallization temperature leads to a high dislocation density inside the austinite, which may increase the nucleates of α and refine the grains in the following laminar flow cooling process. One can also obtain the smaller austinite grain size because of recrystallization by rolling the slab at the temperature higher than the recrystallization temperatuare, which also significantly influences the room-temperature state of the product.

In actual TSCR production lines, the slab generally needs to be rolled up to seven passes. Various phenomena, including the mechanics and heat transfer within the slab and the roll, as well as the metallurgical behavior of the slab, are involved. It is impossible to reveal the effect laws of different processes on products in the laboratory. Cross-scale simulation is a promising approach for uncovering the development of the temperature distribution, stress distribution, strain distribution, dislocation density, and grain orientation during hot rolling, which is beneficial to improving the production process and further saving energy.

### 3.1. Integrated Models on Simulations of the Continuous Hot-Rolling Process

A complete simulation of hot rolling of steel slabs is complex because it involves various phenomena, including mechanics, heat transfer within the slab and the roll, as well as the metallurgical behavior of the slab. There has been extensive research conducted on simulating the hot-rolling process. The FE method has been extensively utilized in the simulation of mechanics and the heat transport phenomena, successfully revealing the flow behavior of metal during rolling [46,47,48,49,50]. The fundamental equation for the FE formulation is represented as:

(6) $$\int_{V}\bar{\sigma }\delta \dot{\bar{\varepsilon }}dV+K\int_{V}{\dot{\varepsilon }}_{V}\delta {\dot{\varepsilon }}_{V}dV-\int_{s}F_{i}\delta u_{i}ds=0,$$

where $\bar{\sigma }$ is the equivalent stress, $\dot{\bar{\varepsilon }}$ is the equivalent strain rate, $F_{i}$ is the surface tractions, $u_{i}$ is the velocity field, ${\dot{\varepsilon }}_{V}$ is the volumetric strain rate, and K is the penalty constant. The heat conduction equation is different from Equation (2), even when disregarding heat radiation, because the heat flux, $\dot{q}$, caused by heat dissipation from plastic work is important, as shown in the following equation:

(7) $$k\nabla^{2}T+\dot{q}-\tilde{\rho }c_{p}\dot{T}=0.$$

The simulation of heat transport in a rolling system is crucial due to the significant temperature variation within the system. When the slab enters the rolling mill, its temperature is higher than that of the roll. Factors such as thermal radiation, air cooling of the roll/slab, water cooling of the roll, and heat produced by the plastic deformation of the slab all contribute to the temperature of the system, ultimately impacting the mechanical properties of the slab. Numerous studies have focused on simulating temperature profiles within the strip and the roll [51,52,53,54,55,56]. Similarly, the thermomechanical behavior of work rolls in rolling mills has attracted significant research interest, primarily for assessing their service life [57,58,59].

During the thermoplastic processes of metals, key microstructural evolution mechanisms include dynamic recrystallization (DRX), meta-dynamic recrystallization (mDRX), static recrystallization (SRX), dynamic recovery (DRV), and static recovery (SRV) [60]. Discontinuous DRX (dDRX) is frequently observed in materials with low stacking fault energy (SFE), whereas continuous DRX (cDRX) is more common in materials with high SFE [61]. Besides, the phenomenon of geometric dynamic recrystallization (gDRX) was also noted during the deformation of aluminum to significant strains at high temperatures [61,62]. The behaviors outlined above are termed as recrystallization phenomena/behavior in this study. Accurately predicting and effectively controlling the recrystallization behavior is crucial for designers seeking to attain exceptional mechanical characteristics of metals and alloys via hot working processes.

Thermomechanical factors, such as temperature, strain, and strain rate, are vital in influencing the recrystallization behavior of metals during hot rolling. Metallurgical equations, i.e., the extended JMAK models [63,64]:

(8) $$f_{\mathrm{rx}}=1-\exp\left(-rt^{n}\right),$$

where *r* represents the nucleation rate and *n* the JMAK exponent, are commonly employed to simulate recrystallization and grain growth, which is convenient to couple with the above FE method, allowing for a thorough examination of the mechanical, thermal, and metallurgical aspects of the hot-rolling procedure. For example, simulations [65,66] were conducted to analyze grain size distributions and the recrystallized fraction within the rolled strip. Further, Wang and Tseng [67] and Jin et al. [68] considered the thermal interaction between the rolls and the strip. Tang et al. [69] and Wang et al. [70] formulated integrated mathematical models to forecast the microstructure evolution of C-Mn steel on the CSP production line and low-carbon steel SS400 during multi-pass hot rolling, respectively.

**Figure 8:**
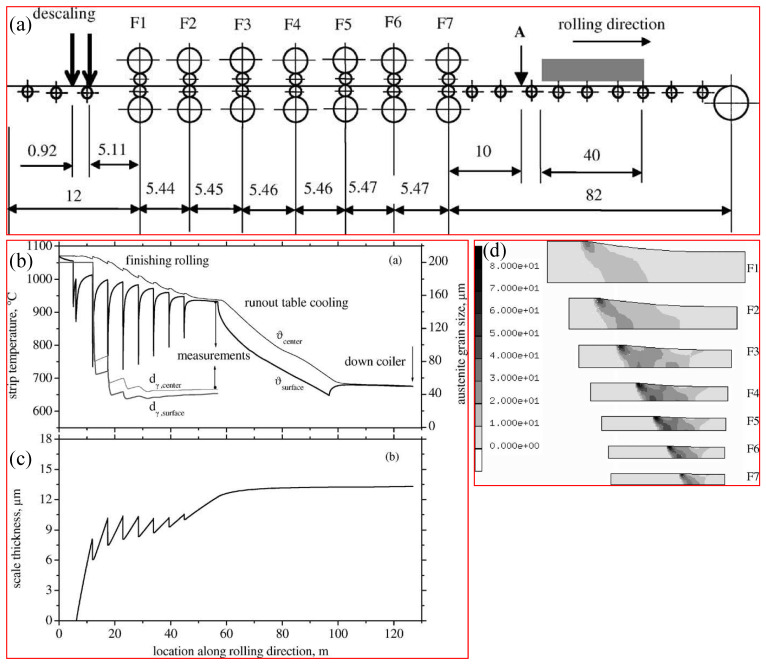
(**a**) Arrangement of a finishing mill consisting of seven stands. Predicted profiles of (**b**) temperature and austenite grain size, and (**c**) scale thickness within a finishing mill. (**d**) Effective strain rate (s^−1^) across various roll gaps from stand Fl to F7. Reprinted with permission from [71].

In 2003, Zhou [71] established a comprehensive FE-based mathematical model capable of predicting the temperature distributions within the strip and work roll, the roll loading, roll longevity, scale thickness on the strip, and the metallurgical attributes of the strip throughout a mill train. It was applied in a seven-stand tandem mill with forecasted temperature, austenite grain size, scale thickness distributions, and strain rate depicted in Figure 8. The 37.5 mm-thick plate underwent seven reduction steps to yield a 9.6 mm strip. As shown in Figure 8b, the temperature at the center of the strip consistently exceeded that of the surface. The strip surface temperature experienced a rapid decline within the roll gaps because of contact with the comparatively cold rolls, followed by a gradual rise as the heat came out from the center before reaching the next stand. The strip center temperature gradually decreased because of heat dissipation through convection and radiation, while it experienced a slight increase within the roll gaps caused by the heat generated from the deformation. The forecasted temperature at location A after stand F7 matched the experimental findings.

Due to the high strain rate and long inter-stand times, mDRX and SRX are concerned. The uneven deformation and temperature distribution across the strip thickness led to a non-uniform microstructure. During the inter-stand (F1–F2) interval, complete recrystallization occurred rapidly, followed by the relatively rapid growth of the austenite grains compared with the subsequent inter-stands because of the higher temperature. In the succeeding inter-stand stages preceding F5, complete recrystallization took place, but the austenite was only partly recrystallized in the inter-stand F6–F7 and after F7 owing to the reduced temperatures. The grain size of the austenite was refined from 200 μm before the initial stand (F1) to 47 μm at the tripe center and 40 μm at the subsurface following the final stand (F7), demonstrating satisfactory agreement with the measurements.

**Figure 9:**
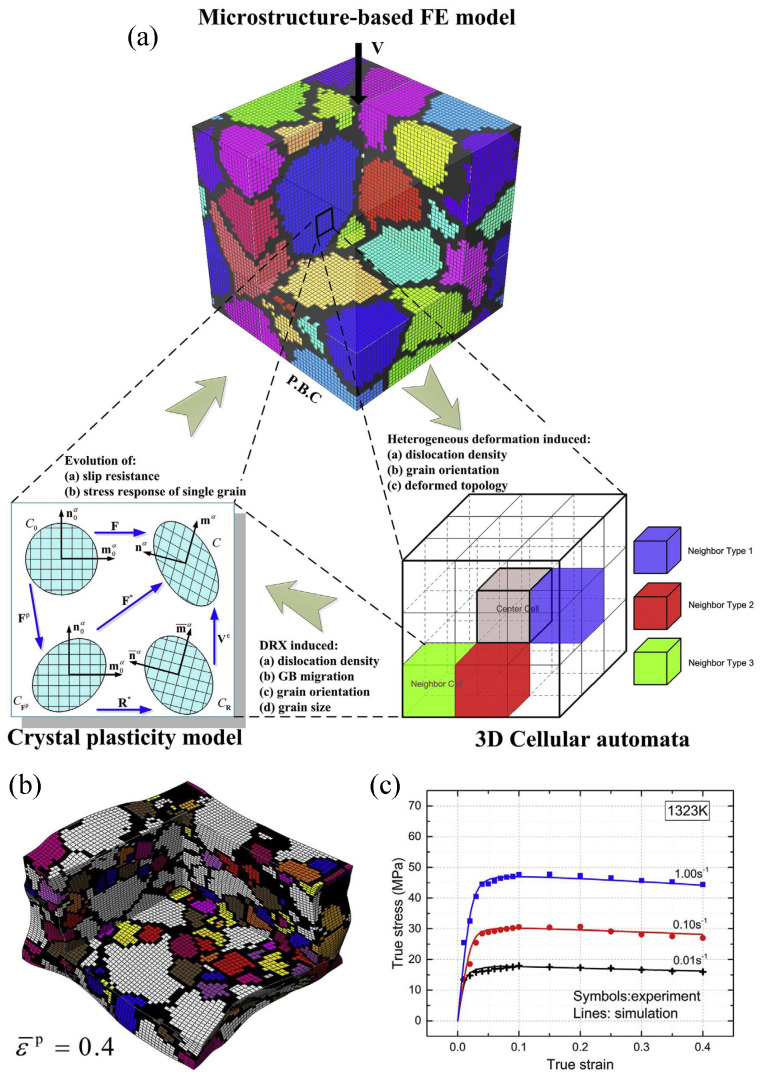
(**a**) Visualization of the 3D-CA-CPFEM model. (**b**) Microstructure with a strain of 0.4 at 1323 K and 0.01 s^−1^. (**c**) Comparison of the simulated and experimental macroscopic stress–strain behaviors of the TA15 titanium alloy subjected to deformation in the bcc phase region at 1323 K. Reprinted with permission from [86].

Generally, the scale thickness increased with time or along the rolling direction, but the rate slowed down, as shown in Figure 8c. It underwent deformation within the roll gap, but the scale thickness did not decrease dramatically due to its limited plasticity. In Figure 8d and Figure 9 of reference [71], the non-uniformity of the strain rate and strain throughout the strip thickness are presented. It is evident that the inhomogeneous plastic deformation within the roll gaps along the thickness and longitudinal directions was pronounced, and the strip surface underwent greater strain and an elevated strain rate relative to the center. Hence, as depicted in Figure 8a, the size of austenite grains remained consistently smaller on the strip surface compared to the center. The zone with the maximum strain rate was situated near the entrance of the roll gap, induced by abrupt changes in rolling speed along the vertical direction.

### 3.2. Mesoscopic Recrystallization Simulations

As presented above [71], the FE method, coupled with the extended JMAK model, can effectively simulate the hot-rolling process. The FE method adeptly captures mechanical and heat transport phenomena [4]. As for the metallurgical phenomena, the extended JMAK model provides a straightforward yet widely used framework for describing recrystallization dynamics. Nonetheless, it cannot depict intricate details, such as the orientation of recrystallized grains, and heterogeneous deformation and nucleation in real cases, etc. [63]. Moreover, to characterize various events, such as DRX, mDRX, SRX, and grain growth, distinct equations are required, resulting in certain discontinuities in transitions between different events [72].

Discrete and continuum morphology-tracking methods, such as CA, PF, MC, Level Set (LS), and Vertex models, are capable of capturing changes in grain morphology. Hallberg [23] provided a review discussing the strengths and weaknesses of CA, PF, MC, Vertex, and LS models for SRX. The fundamental formulas of MC, CA, and PF methods can also be found in Section 4. To elucidate the progression of dislocation density during deformation, the following Kocks–Mecking (KM) model is frequently coupled with morphology-tracking models, but it fails to describe the heterogeneous deformation [73]:

(9) $$\frac{d\rho_{i}}{d\varepsilon }=k_{1}\sqrt{\rho_{i}}-k_{2}\rho_{i},$$

where $\rho_{i}$ represents the dislocation density of grain i, ε is the true strain, $k_{1}$ is a constant representing hardening, and the coefficient of the DRV term $k_{2}$ is the function of temperature and strain rate. The driving force of grain growth caused by the difference in stored energy between grains i and j can be obtained as:

(10) $$\Delta E_{ij}=\frac{1}{2}\mu b^{2}\left(\rho_{i}-\rho_{j}\right),$$

where μ is the shear modulus and b is the magnitude of the Burgers vector. Crystal plasticity (CP) models, notably those based on dislocation density, as demonstrated in [74,75,76], offer a valuable framework for describing the morphological characteristics of DRX and capturing the variation in deformation and texture at the mesoscale. The work of Li et al. [77], on CP modeling applied to DRX, describes the heterogeneous deformation at the grain level, the orientation of grains, and the mechanical response of a single crystal. Thus, an effective approach to model the microstructure evolution of recrystallization involves combining the aforementioned morphology-tracking models with CP models. The most extensively studied coupling is between CA and CP models [60], with the coupling of PF and CP models also receiving attention. Recrystallization is a prevalent occurrence in metals subjected to deformation, irrespective of the processing techniques used. Therefore, modeling and simulation approaches for recrystallization in other materials (e.g., copper and aluminum) can also be applied to steels produced by conventional or TSCR techniques. Hence, this section offers a concise summary of the applications of CA and PF methods in recrystallization. Readers interested in the methods of coupling CP with LS and Vertex methods can refer to [78,79] and [80], respectively.

#### 3.2.1. Application of the Cellular Automata Method in Recrystallization Simulations

The CA method is widely used in modeling recrystallization, as evidenced by its applications in reviews on SRX [23,81,82] and DRX [60]. Hence, we provide a concise review here. Raabe and Becker [83] pioneered the integration of a crystal plasticity finite-element (CPFE) model with a probabilistic CA to imitate SRX in aluminum, in which the deformation outcomes derived from the CPFE model serve as inputs for calculating SRX via the CA method. Later, Popova et al. [84] formulated a microstructure-informed CPFE model, wherein the grids within the FE model were utilized by the CA model for 2D DRX calculations, thus achieving a synchronized response between mechanical and microstructural evolution. This model addressed the problem of the absence of grain orientations being transferred from crystal plasticity finite element method (CPFEM) elements to CA cells, which occurs in the model developed by Wu et al. inspired by Raabe and Becke [83,85].

Li et al. [86] proposed a three-dimensional cellular automata-CPFEM (3D-CA-CPFEM) model, which considers the changes in morphology during DRX as an inherent aspect of material constitutive behavior. This integration is achieved by integrating the 3D semi-probability CA approach into the CPFEM architecture. This framework enables comprehensive predictions of macroscopic shaping, mesoscale deformation mechanisms, and microscale microstructural changes. Figure 9a presents the 3D-CA-CPFEM model, showcasing both the finite elements and CA cells. Utilizing the CPFEM model enables the comprehensive capture of heterogeneous deformation phenomena occurring both at the macroscale (grain aggregates) and at the mesoscale (individual grains and slip systems). This capability is particularly effective in representing material heterogeneity and varying grain orientations, as shown in Figure 9b. As a result, the model can provide insights into the non-uniform distribution of dislocation density, slip resistance, and stress response within each grain, considering the evolution of dislocation density during multiscale heterogeneous deformation [86]. The calculated dislocation density, orientation, and deformed topology of individual grains are utilized in the CA method to calculate the evolution of DRX. In Figure 9c, the predicted flow stress of the representative volume element and the kinetics for DRX are contrasted with experimental data from the isothermal compression tests of the TA15 titanium alloy in the body-centered cubic (bcc) phase region at 1323 K [86].

It is worth noting that methods coupling DRX and CP models [86] can readily be applied in SRX or mDRX simulations as well [87].

#### 3.2.2. Application of the Phase-Field Method in Recrystallization Simulations

While the application of the PF method in modeling recrystallization is less common compared to the CA method, it remains an important approach [23]. We will offer a concise overview of PF models applied to recrystallization simulations, primarily focusing on those reported after the aforementioned review [23].

The PF model for SRX has been integrated with various physical mechanisms and utilized in diverse materials, such as low-carbon steel [88], ultra-low carbon steel [89], titanium alloy [90], and C–Mn steel [91]. Takaki and Tomita [92] implemented the SRX simulation by utilizing the deformed microstructure obtained from the integration of the multi-phase-field (MPF) method and the FE method based on the CP model as the initial microstructure. Subsequently, Takaki et al. formulated a DRX model through the integration of the MPF model and KM model [93,94], which was applied to deforming tungsten by Li et al. [95]. Further, Takaki et al. [96] expanded their research by substituting the flow stress model with an FE model incorporating elastic–plastic characteristics, aiming to develop a fully coupled model. Recently, Cai et al. [97] devised a PF model for dDRX to simulate the hot deformation of magnesium alloys. However, the aforementioned DRX models assume the mean dislocation density within each grain due to the application of the KM model [73].

**Figure 10:**
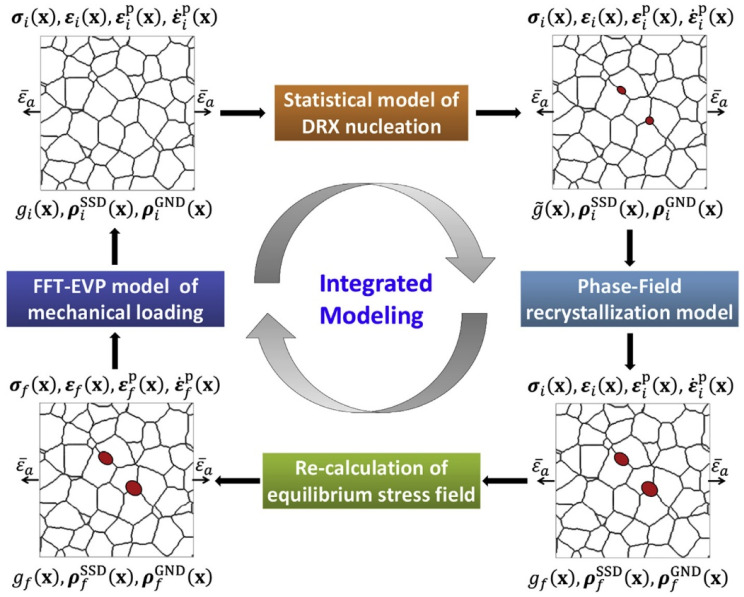
Diagram depicting the integrated modeling approach employed to simulate DRX. Reprinted with permission from [102].

Hiebeler et al. [98,99,100] developed a novel simulation framework for modeling microstructure evolution and concurrent flow stress during rolling processes. This approach couples the CP model with recovery, recrystallization, and grain evolution kinetics within a PF framework. Simulation of the macroscopic flow curve evolution, as well as 2D and 3D microscopic grain structure, exhibit strong consistency with the measurements from hot compression and double-hit compression tests. Zhao et al. [101,102] developed an integrated 3D model that tightly integrates a fast Fourier transform-based elasto-viscoplastic model with a PF model. This integration includes the combination of a CP model and a stochastic model of DRX nucleation, employing the grain boundary bulging mechanism [103,104]. This model was applied to investigate the evolution of microstructural and micromechanical fields during uniaxial compression of polycrystal copper at different high temperatures [102]. The basic integration scheme is presented in Figure 10. It should be noted that the elastic energy is considered in the work of Hiebeler et al. [98,99].

### 3.3. Cross-Scale Simulations

The coupling of CA or PF methods with the CP model enables modeling of the recrystallization during the hot-rolling process, but it is time-consuming, particularly for the 3D DRX simulation. For instance, the model developed by Li et al. [86] achieved complete integration of multiscale heterogeneous deformation, mechanics, and microstructural evolution. Consequently, if the simulation space is expanded to the macro-scale, the model has the potential to conduct macroscopic simulations. Nevertheless, due to the extensive computational requirements associated with simulating microstructure evolution using a very fine mesh, the simulation was conducted within a space measuring 50 × 50 × 50 μm [86].

To realize cross-scale simulation, it is necessary to integrate the above methods with the macroscopic FE method. For instance, Wu et al. [105] implemented the DRX simulation of 2.25 Cr-1 Mo-0.25 V steel by integrating the CA and FE models. They utilized the time-saving KM model and implemented the simulation within a relatively small 2D macroscopic space of 5 mm × 15 mm, resulting in acceptable computation time despite mapping the FE and CA spaces. Capturing mesoscale heterogeneous deformation and texture evolution in a large 2D (or even 3D) space poses challenges in achieving complete mapping of macroscopic simulation space to microscopic space due to significant computational demands.

**Figure 11:**
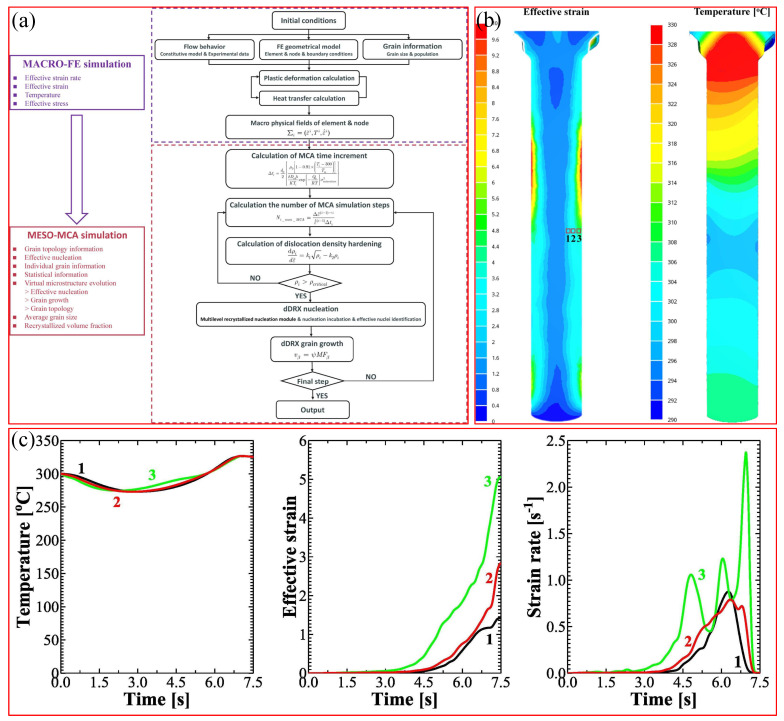
(**a**) Multiscale modeling approach flowchart. (**b**) Simulated temperature and strain using the PF method within the deformed billet. Regions delineated by the red boxes labeled from 1 to 3 denote the region designated for the MCA simulation. The characterization sites, moving from right to left, are at distances of 0.25 mm, 1 mm, and 3 mm from the billet surface. (**c**) FE calculation of the temperature, effective strain, and strain rate of the examined element nodes within the deformed billet. Reprinted with permission from [106].

One approach to deducing the computation time for cross-scale simulation in a large space is to conduct macroscopic simulation first, followed by selecting several representative microscopic regions for recrystallization simulation. For example, Chen et al. [106] constructed a multiscale integration model by integrating the CA and FE methods, to simulate the dDRX by improving the multilevel CA model by Chen et al. [107]. The model was validated through the macroscopic extrusion experiment. As depicted in Figure 11, the FE simulation enabled the acquisition of spatial distributions of temperature, stress, strain, and strain rate over time, which were mapped to the CA simulation to reveal the microstructure evolution in different areas selected based on practical requirements. However, a limitation of this method lies in the absence of feedback from microsimulation to macrosimulation. Should the macroscopic material properties be significantly influenced by the microstructure evolution, this method could potentially introduce considerable errors.

## 4. Cross-Scale Simulation of the Laminar Flow Cooling Process

Laminar flow cooling is an important process for the structural performance of TSCR production lines. Experience has shown that various strip steel products can be achieved through judicious application and optimization of the laminar flow cooling process, even with identical steel compositions. During laminar cooling, γ→α transformation takes place as the thin plate temperature decreases, serving as a pivotal factor in dictating the microstructure and mechanical characteristics of steel materials in their utilized state [108,109]. Thus, a deep comprehension of the evolution process of the microstructure during steel cooling is essential for product development and controlling structural performance in new strip products.

With advancements in computer technology, material structure simulation techniques are becoming more sophisticated. Presently, simulations of the cooling transition process incorporate methods such as MC, CA, and PF. By integrating multiple simulation approaches, cross-scale simulations of sheet microstructure can be achieved, thereby improving our understanding of microscopic steel structure evolution during cooling.

### 4.1. Microscopic Cooling Phase Transition Simulations

#### 4.1.1. Application of the Monte Carlo Method in Cooling Phase Transition Simulations

In the MC method, microstructure evolution and carbon concentration field progression are predominantly conducted through sampling techniques. The influence of interface energy and carbon diffusion on system evolution is accounted for by both the interface energy term and the chemical free energy term of the Hamiltonian operator, which can realize the structure simulation of austenite ferrite diffusion transition while low-carbon steel undergoes continuous cooling on a moderate scale [110].

The chemical free energy term and the interface energy term are introduced into the Hamiltonian of the system in the Q-state Potts model [111,112]:

(11) $$H=\frac{1}{2}\sum_{i}^{n}\sum_{j}^{m}G_{S_{i}S_{j}}^{b}+\sum_{i}^{n}G_{i}^{c}$$

where $G_{i}^{c}$ is the chemical free energy of cell *i* with orientation *S_i_*, and $G_{S_{i}S_{j}}^{b}$ is the interface energy between MC cell *i* and cell *j* with different orientations.

For MC simulations, it is difficult to convert the time scale to the real time because of the random transition attempt in the Q-state Potts model. According to the rate theory of the time scale proposed by Raabe [113], the relations between MC steps (MCS) and real time can be built qualitatively using the phenomenological model of grain boundary motion, as follows:

(12) $${\dot{X}}_{\mathrm{MC}}={\dot{X}}_{\mathrm{rate}}$$

i.e.,

(13) $$\frac{\lambda_{\mathrm{MC}}}{\Delta t_{\mathrm{MC}}\Delta t_{\mathrm{real}}}=m_{i}p_{i}$$

Here, ${\dot{X}}_{MC}$ is the boundary velocity of the MC model and ${\dot{X}}_{\mathrm{rate}}$ is the boundary velocity according to the phenomenological model of boundary motion. *m_i_* is the local grain boundary mobility and $p_{i}$ is the local driving force of boundary motion. Δ*t*_MC_ is the kinetic MC measure, in units of MCS, λ_MC_ is the jump width or lattice parameter of the Potts model, in m, and Δt_real_ is the real time step, in s/MCS. According to Equation (13), the concise relation between MCS and real time can be built.

**Figure 12:**
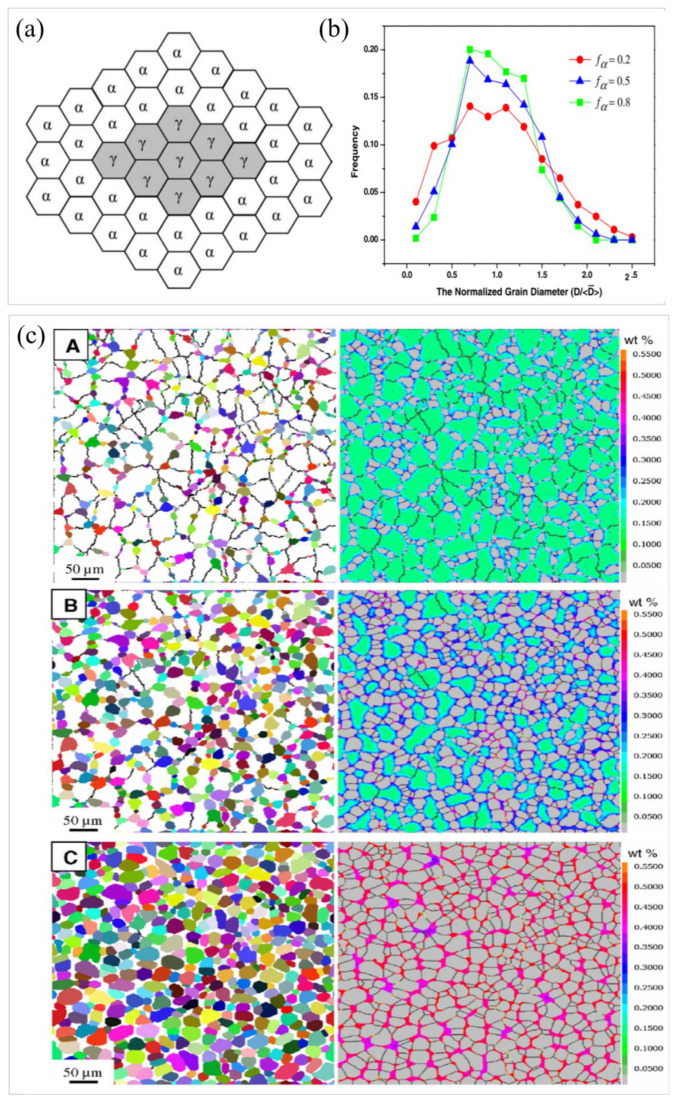
(**a**) Diagrammatic representation of the hexagonal grid employed for depicting the binary phase system [110]. (**b**) Standardized distributions of ferrite grain sizes modeled by the MC model. (**c**) Modeled comprehensive transformation dynamics employing the MC model. (**c**) (**A**–**C**) depict the temporal progression of the microstructure (**left**) and the carbon concentration distribution (**right**) throughout the transition. Reprinted with permission from [111].

To study the development patterns of ferrite grains during the phase transformation process, Li et al. [112] implemented the microstructure evolution simulation during the phase transformation process using the MC model and analyzed the size distribution of ferrite grains at different phase transformation stages. The microstructural arrangement was projected onto a 2D hexagonal lattice, as shown in Figure 12a. Every hexagonal cell could be assigned to either the ferrite or austenite phase. There were three parameters used to characterize the state of an MC cell *i*, i.e., the order parameter *η_i_*, the carbon concentration *C_i_*, and the orientation *S_i_*. The normalized distribution of ferrite grain sizes at various transformation stages is depicted in Figure 12b. The distribution of all grains in different phases of phase transition after normalization satisfies the relation: $\sum \varrho \left(x\right)dx=1$, with $\varrho \left(x\right)$ and x as the size distribution and the normalized diameter, $D_{i}/\bar{D}$, respectively. Simulation results indicated that the interaction of ferrite nucleation, growth, and coarsening led to a more inhomogeneous grain size distribution at the onset of the phase transition, making the distribution peaks at the later stages of the phase transition narrower than those at the beginning. At the initiation of the phase transition, fine ferrite was continuously nucleated at the grain boundaries, and most of the ferrite grains were able to grow into the austenite interior. Therefore, the early nucleation time affected the size of the ferrite grains at the later stage. During the latter stages of phase transition, ferrite grains grew along the austenite grain boundaries and collided with each other. As the phase transformation proceeded, the boundaries of the austenite grains were depleted by ferrite grains and the effective position of ferrite nuclei decreased. The enlargement of adjacent ferrite grains became a significant mechanism influencing the size of ferrite grains. Since the larger grains possess reduced interfacial curvature, the larger ferrite grains would engulf their adjacent smaller ferrite grains, which resulted in a narrower ferrite size distribution in the later stages. The results of the development of the microstructure and the carbon concentration distributions with time throughout the phase transition modeled by the MC model are presented in Figure 12c. The evolution pattern of the structure diagram also provided a good indication of the outcomes of normalizing the distribution of grain sizes. From the concentration distribution diagram, it was observed that at the position of high nucleation density, carbon atoms were biased at the advancing phase transition interface, thereby reducing the driving force for interfacial migration. This phenomenon resulted in smaller ferrite grain sizes.

#### 4.1.2. Application of the Cellular Automata Method in Cooling Phase Transition Simulations

**Figure 13:**
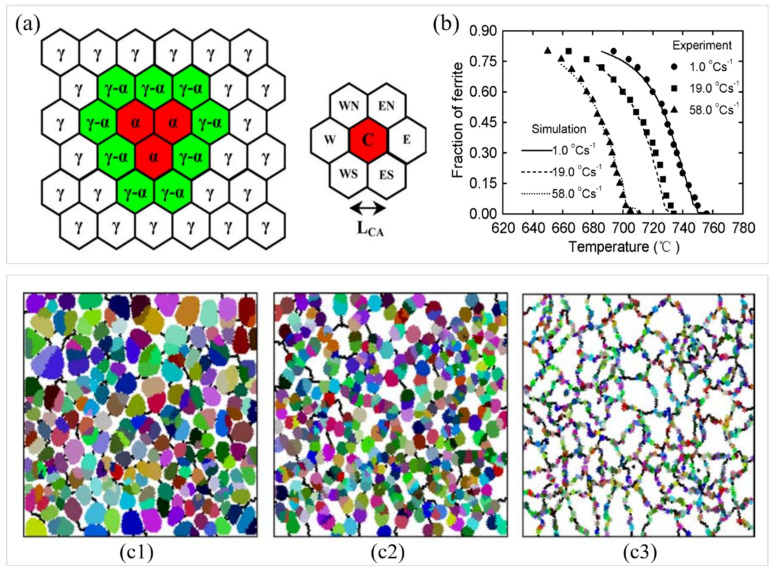
(**a**) Diagrammatic representation of the hexagonal grid within the CA model (**left**) and the vicinity of a cell (**right**). (**b**) Predicted ferrite fractions compared with experimental results [7] in low-carbon steel at cooling rates (*Q*) of 1.0, 19.0, and 58.0 °C/s. The simulated microstructures within a scope of 0.2 × 0.2 mm^2^ at 694 °C under different cooling rates: (**c1**) 1.0 °C/s, (**c2**) 19.0 °C/s, and (**c3**) 58.0 °C/s. Reprinted with permission from [117].

The CA model is capable of simulating the spatial–temporal development of intricate systems. Recently, the CA model has been utilized to simulate solid-state transformations in steels, such as austenitization [114] and the austenite decomposition into ferrite in steel [7,115,116]. As shown in Figure 13a, at time *t*, the growth length, $l_{i,j}^{t}$, of a ferrite cell (*i*, *j*) toward one (*k*, *l*) of its neighboring interface cells can be described as follows [117]:

(14) $$l_{i,j}^{t}=\int_{t_{0}}^{t}v_{i,j}dt$$

where *t*_0_ represents the time at which cell (*i*, *j*) nucleates, and $v_{i,j}$ is the growth velocity of cell (*i*, *j*) toward one (*k*, *l*) of its neighboring interface cells, which depends on the local carbon concentration in austenite and ferrite sides at the *γ-α* interface. At time *t*, the fraction, $f_{k,l}^{t}$, of ferrite phase in an interface cell (*k*, *l*) resulted from the growth of its neighboring ferrite cell (*i*, *j*) can be calculated by:

(15) $$f_{k,l}^{t}=\frac{l_{i,j}^{t}}{L_{\mathrm{CA}}}$$

where *L*_CA_ is the length of a cell in the CA simulation. The fraction, $f_{k,l}^{t}$, of ferrite phase in cell (*k*, *l*) at time *t* is the sum of the fractions of ferrite resulted from all its neighboring ferrite cells.

To examine the competitive dynamics between the nucleation and initial expansion of austenite and ferrite phases, Kumar et al. [115] employed a CA algorithm to model the phase change from austenite to ferrite. The simulation outcomes matched the experimental findings studied by Militzer et al. [118], particularly concerning the changes in the phase transformation onset temperature, austenite grain number, and the size of ferrite grains relative to the cooling rate. To study the comprehensive and complex behavior of ferrite manager nucleation and growth throughout the phase transition in mild steel, Zhang et al. [116] formulated a 2D CA model. The simulation results showed that larger cooling rates promoted the formation of finer ferrite grains, aligning with the experimental results.

For investigating the phase change from austenite to ferrite during the gradual cooling of mild steel, Lan et al. [117] utilized a CA approach to model the phase transition process at three cooling rates and found that the proportion of the formation of newly created ferrite varied with the cooling rate. The CA model is shown in Figure 13a, left. The hexagonal cells in the system denoted specific properties of the state of each cell. The transformation was performed for every cell based on the transformation principles. The conversion principles of state variables in CA simulations were gained by grain nucleation and growth models. The six nearest cells were defined around each cell, as shown in Figure 13a. For modeling the phase transition from austenite to ferrite, each cell in the lattice had six variables: a crystal orientation variable, indicating the crystallographic orientation of the ferrite nuclei; a phase fraction variable, indicating the phase fraction of ferrite; a phase state variable, implying whether the lattice position belongs to the γ phase, the α phase, or the α-γ interface; finally, three concentration variables, indicating the carbon concentration in austenite, the carbon concentration in ferrite, and the average carbon concentration. Figure 13b shows the proportion of ferrite volume in mild steel during phase transition at different cooling rates measured through simulation and experimentation. The simulated outcomes closely matched the experimental findings, except for marginally lower ferrite volume fractions observed at 19.0 °C/s at the onset and 58.0 °C/s at the end of the phase transition. At the three cooling rates, 1.0, 19.0, and 58.0 °C/s, the simulated final ferrite fractions were 80%, 74%, and 73%, respectively, while the experimental outcomes yielded approximately 80%. The significant deviation between simulated outcomes at 19.0 C/s and experimental results at 58.0 °C/s was attributed to the simulated completion temperature being higher than the experimental one. At cooling rates of 19.0 and 58.0 °C/s, the simulated finishing temperatures were 680 and 658 °C, respectively, while the experimental outcomes were 664 and 650 °C, respectively. The deviation could stem from underestimating the carbon diffusion rate in austenite, resulting in a higher simulated carbon concentration at the γ-α interface compared to the experimental data. This might also be ascribed to the neglect of carbon diffusion dynamics and interface mobility of *γ*-*α* along the grain boundaries of austenite in the applied CA model. In addition, it could also be seen that the mean rate of ferrite growth exhibited a gradual decrease throughout the continuous cooling process in Figure 13b. Figure 13(c1–c3) illustrates the microstructure at 694 °C at different cooling rates.

#### 4.1.3. Application of the Phase-Field Method in Cooling Phase Transition Simulations

Currently, some important progress has been made in describing the dynamics of the transition from austenite to ferrite by using the PF method [5,119,120,121,122,123]. Here, we took the MPF model [124] as an example to present the model. The whole energy is the integration of the energy density functional over the domain *V*. It comprises three main components, namely, the chemical free energy density, *f*_chem_, interfacial free energy density, *f*_int_, and elastic free energy density, *f*_elast_:

(16) $$F=\int_{V}\left(f_{\mathrm{int}}+f_{\mathrm{chem}}+f_{\mathrm{elast}}\right)dV$$

For a system with *n* phases and *m* components, the multiphase-field equations are derived from the kinetic equation:

(17) $$\frac{\partial \phi_{\alpha }}{\partial t}=-\sum_{\beta =1}^{\nu }\frac{{\tilde{M}}_{\alpha \beta }}{\nu }\left(\frac{\delta F}{\delta \phi_{\alpha }}-\frac{\delta F}{\delta \phi_{\beta }}\right)$$

with interface mobilities ${\tilde{M}}_{\alpha \beta }$, the phase-field variables $\phi_{\alpha }$, and the number of phases ν which is locally coincident. The diffusion equation is:

(18) $$\frac{\partial c^{k}}{\partial t}=\nabla \cdot \left(\sum_{\alpha =1}^{n}\sum_{i=1}^{m-1}\phi_{\alpha }D_{\alpha }^{ki}\nabla c_{\alpha }^{i}\right)$$

with the interdiffusion coefficient $D_{\alpha }^{ki}$, the mixture composition of *k* component *c^k^*, and the composition of *k* component in *α* phase $c_{\alpha }^{k}$.

**Figure 14:**
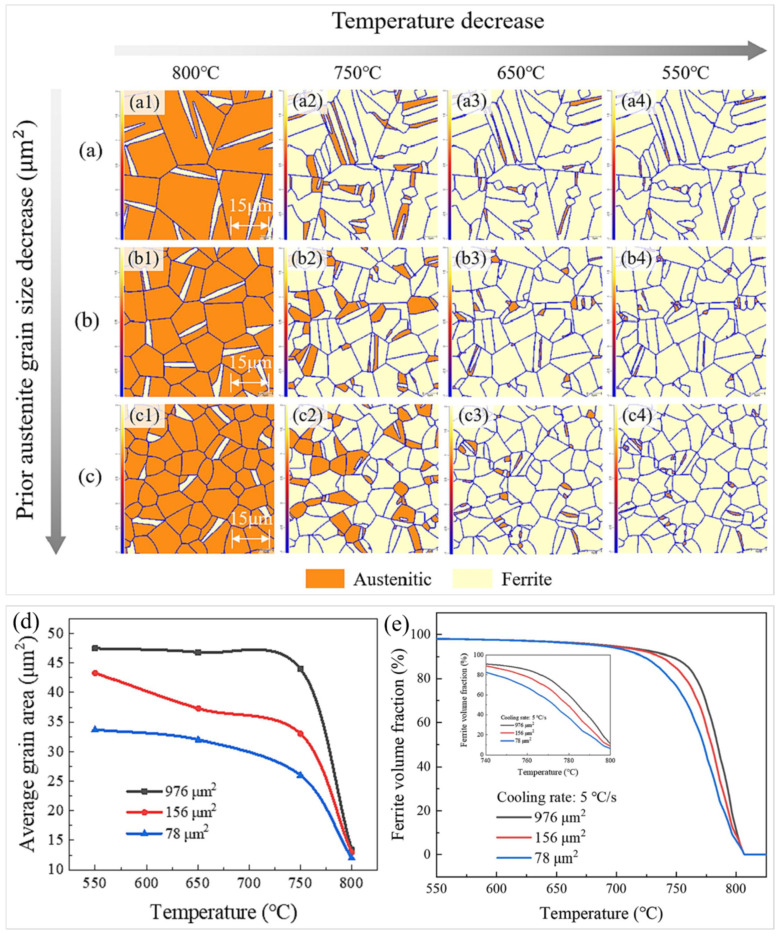
The simulated evolution of microstructures transitioning from austenite to acicular ferrite, characterized by a cooling rate of 5 °C/s, a nucleation density of 3.84 × 10^−3^ μm^−3^, and various PAGS, with phase transition temperatures ranging from 800 °C to 550 °C: (**a**,**a1**–**a4**) 976 μm^2^, (**b**,**b1**–**b4**) 156 μm^2^, and (**c**,**c1**–**c4**) 78 μm^2^. (**d**) The simulated average grain area of acicular ferrite concerning temperature at different PAGS. (**e**) The variation in acicular ferrite volume fraction concerning temperature and diverse PAGS at a cooling rate of 5 °C/s. Reprinted with permission from [125].

Yeon et al. [119] employed the PF method to investigate the α-γ quasi-equilibrium transition in the Fe-Mn-C system. However, the application of the PF method to explore the phase transition of acicular ferrite in a polycrystalline microstructure is rarely reported. To shed light on the effect of the pristine austenite microstructure on acicular ferrite in low-alloy steels, Lv et al. [125] employed the PF method to simulate the phase transition microstructure at various prior austenite grain sizes (PAGS), which was found to affect both the ferrite volume fraction and grain size. Figure 14a–c represent the microstructures of PAGS for 976, 156, and 78 μm^2^ with decreasing temperatures, respectively. As the PAGS decreased, the proliferation of austenite grain boundaries occurred, thereby impeding the growth of acicular ferrite and contributing to grain refinement. The mean dimension of the acicular ferrite increased gradually as the cooling rate decreased, with the tiniest acicular ferrite observed at a PAGS of 78 μm^2^ within the studied continuous cooling temperature range, as shown in Figure 14d. In Figure 14e, the proportion of acicular ferrite among various PAGS at a cooling rate of 5 °C/s is presented. It indicated that the larger PAGS at the beginning of the phase transition resulted in a higher proportion of acicular ferrite. Upon a further reduction in temperature to 550 °C, the proportion of acicular ferrite eventually equalized by the conclusion of the phase transformation. Due to the unavoidable presence of residual austenite in this investigation, the ferrite fraction in the ultimate phase transformation structure did not reach 100%.

### 4.2. Cross-Scale Simulations

**Figure 15:**
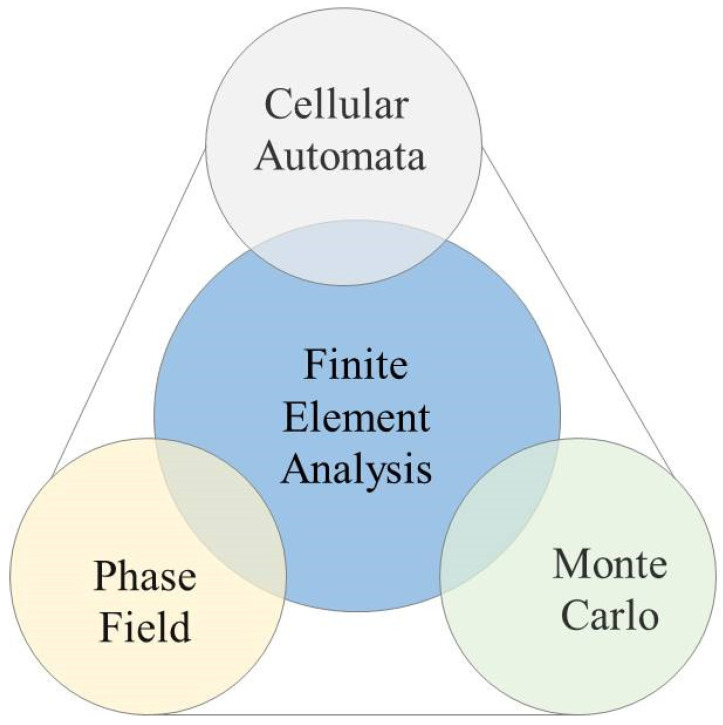
Coupling diagram between macroscopic FEA and microscopic simulation methods.

Similar to the casting and hot-rolling processes, the MC, CA, or PF microscopic simulation methods cannot be scaled up directly to macroscopic simulation applications of the laminar cooling process due to their extensive computational demands. Integrating macroscopic FEA with microstructure simulation methods facilitates cross-scale simulation of materials in the laminar cooling process, as depicted in Figure 15. FEA provides effective boundary conditions for microscopic simulations, which in turn deliver detailed microstructure information of the sheet material back to the FEA framework.

Chen et al. [126] implemented cross-scale modeling of steel during cooling by integrating the CA method and FEA. The study concentrated on resolving the strain, strain rate, and temperature distributions within hot-rolled steel plates over multiple rolling passes via FE simulations. The outputs from these simulations were subsequently input into a CA model to simulate grain coarsening during both the heating and the ensuing cooling processes. Meanwhile, the transformation of deformed austenite to ferrite was studied by Yamanaka et al. [127] by coupling the CPFE method with the MPF method. This model pinpointed the location and density of ferrite nucleation within deformed austenite using the CPFE method, while the nucleation rate of ferrite at each site was determined utilizing classical nucleation theory. Subsequently, MPF simulations were employed to explore how phase transformation kinetics are influenced by plastic deformation in the austenitic phase and holding temperature. It is noteworthy to mention that this process may also involve the formation and growth of precipitated phases [128]. In conclusion, a more comprehensive understanding of the laminar cooling process can be achieved by integrating simulation methods across multiple scales.

## 5. Discussion

Under the background of carbon neutrality, numerical simulation of the TSCR process, mainly involving casting, hot-rolling, and laminar flow cooling processes, has become an increasingly important research method. It is important to clarify the basic characteristics, including the advantages and disadvantages of each simulation method, and to choose the appropriate research method to realize the simulation of three processes.

### 5.1. Cross-Scale Simulation of the Continuous Casting Process

The MC method, an early approach to simulating solidification microstructure evolution, is relatively simple and does not account for the influence of solidification time, leading to lower accuracy and persuasiveness. However, it has been significantly refined and developed over the years, serving as a valuable reference for other numerical simulation methods. The relatively new PF and CA methods stand out as the two most extensively employed simulation techniques for solidification structures [3,8,11,16]. By utilizing the CA method in solidification studies, researchers can gain valuable insights into the formation of microstructures in materials, such as steel. This approach facilitates the optimization of processing parameters, the estimation of material characteristics, and the development of innovative alloy designs tailored for specific applications. As computational capabilities advance, the CA method continues to stand out as a valuable tool for deepening our understanding of solidification phenomena in metallurgy. Despite its computational demands, the capacity of the PF method to capture intricate microstructural features and dynamic phase transformations makes it a valuable tool for both fundamental research and industrial applications in understanding and optimizing solidification processes. Ongoing advances in numerical methods and high-performance computing further enhance the precision and effectiveness of the model, paving the way for continued exploration and innovation in the field of solidification science.

The three aforementioned simulation methods are only applicable at the microscopic scale. Concerning cross-scale applications, the CA-FE/FD coupling model is frequently employed in conjunction with the actual production processes of continuous casting. It overlooks the non-uniformity of the actual solidification process, treats the large billet symmetrically, and skips numerous calculations, resulting in a broad calculation scope, high efficiency, and extensive utilization in continuous casting process research and optimization. With CA-FE models integrated into ProCAST, beginners can save time on programming and testing, allowing them to concentrate more on CA applications and creative endeavors.

### 5.2. Cross-Scale Simulation of the Hot-Rolling Process

The FE method coupled with the extended JMAK model offers a comprehensive integration of various phenomena, including heat transport and mechanics in the strip and the roll, and the metallurgical behavior of the strip, which can effectively simulate the hot-rolling process [71]. However, the extended JMAK model cannot capture detailed features, such as the orientation of recrystallized grains [63]. To achieve multi-scale heterogeneous deformation simulation in the hot rolling of metallic engineering materials, it is necessary to utilize morphology-tracking methods (such as CA and PF methods) coupled with the CP model [86,129]. Nevertheless, the coupling model necessitates extensive computation with a fine mesh to simulate microstructure evolution, rendering it challenging to conduct simulations over large spatial scales at present [86]. With the ongoing advancement of computational power, achieving this may become feasible in the future.

To reduce the computation time of cross-scale simulation for a large space, one approach is to conduct a macroscopic FE simulation first, followed by selecting several representative microscopic regions for recrystallization simulation [106]. If DRX is absent or negligible, this approach proves effective. However, the situation grows more complex when deformation and DRX interact. Without feedback from microsimulation to macrosimulation, significant errors may arise if the macroscopic material properties are significantly influenced by microstructural evolution due to DRX. In such scenarios, updating macro-model parameters based on microsimulation results becomes necessary during cross-scale simulation. Additionally, a greater number of representative microscopic recrystallization simulation regions are required when uneven deformation is more prevalent.

### 5.3. Cross-Scale Simulation of the Laminar Flow Cooling Process

The three simulation methods we discussed in Section 4, namely, MC, CA, and PF methods, can effectively simulate the microscopic structure during laminar cooling. However, each simulation method has its characteristics tailored for specific application scenarios. The MC model has the advantages of being less restricted by geometric conditions, having a convergence rate unaffected by problem dimensionality, and featuring a straightforward program structure for straightforward implementation. At the same time, the MC model is challenged by a slow convergence rate and stochastic errors. The CA model can integrate local concentration variations in nucleation or growth functions, its utilization of hexagonal grids enables a more accurate simulation of isotropic phenomena, and the model is more natural and real. Through integrating the phase, solute, temperature, and other external fields, the model adeptly merges micro- and macro-scales. However, the CA model lacks convenience in accounting for the influence of interfacial energy on phase transitions, and hexagonal grids are difficult and complex to represent and display. The PF model adeptly handles solute aggregation and second-phase precipitation along grain boundaries without necessitating the tracing of grain boundary locations. However, the application of the PF model is limited due to computational demand and the challenge of determining model parameters. In addition, the MC, CA, or PF microscopic simulation methods cannot be scaled up to macroscopic simulation applications due to their extensive computational demands. The integration of macroscopic FEA with microstructure simulation methods has been deemed essential to achieve cross-scale simulation of the laminar cooling process.

## 6. Conclusions and Outlook

The modeling techniques for the coupling of heat transfer, nucleation, grain growth, and ripening have been developed based on classical solidification theory and mathematical approaches, such as the CA-FD/FE method. Numerous modeling tools have been developed, ranging from commercial packages, such as ProCAST, to open-source packages, such as OpenPhase [130]. All of them are focused on general casting and solidification scenarios and simple calculation domains. More complex casting processes, such as high-pressure die casting, electroslag remelting, and extrusion casting processes, need to be considered and addressed in future cross-scale modeling studies. Although the modeling of casting and solidification processes in steel continuous casting process has been researched extensively, due consideration needs to be paid to the proper selection and determination of boundary conditions based on the actual industrial processes, spanning from macro-scale and meso-scale to micro-scale.

The FE method coupled with the extended JMAK model can effectively simulate the hot-rolling process, while the JMAK model cannot capture detailed features, such as the orientation of recrystallized grains. Utilizing morphology-tracking methods (such as CA and PF methods) coupled with the CP model can achieve multi-scale heterogeneous deformation simulation of materials. But a large demand of computation renders it challenging to conduct simulations over large spatial scales at present. One approach to reduce the computation time of cross-scale simulation is to conduct a macroscopic FE simulation, followed by selecting several representative microscopic regions for recrystallization simulation. However, significant errors may arise if the macroscopic material properties are heavily influenced by microstructural evolution caused by DRX, due to a lack of feedback from microsimulation to macrosimulation. By coupling multiple physical fields, such as the temperature field, composition field, and strain field, the microstructural evolution process during laminar cooling can be simulated using PF and CA methods. Further combining macroscopic simulation methods, such as the FE method, enables successful cross-scale simulation of the laminar cooling process.

Cross-scale simulation of casting, hot-rolling, and laminar cooling processes offers theoretical support for optimizing and regulating process parameters in the TSCR process, notwithstanding some unresolved issues. Considering the high computational demands of simulations, particularly for microscopic simulations, it is imperative to explore methods to expedite the process: (1) advancements in computing power can speed-up the simulations, (2) when integrated with a material microstructure database, data-driven simulation methods utilizing machine learning models may significantly enhance computational efficiency, and (3) a prospective PF method, known as the sharp-phase field method, was recently introduced by Finel et al. [131], wherein interfaces are resolved numerically using essentially a single grid point, significantly improving efficiency [132].

## Data Availability

Not applicable.
